# Local ecological knowledge and folk medicine in historical Estonia, Livonia, Courland, and Galicia, 1805-1905

**DOI:** 10.12688/openreseurope.14406.1

**Published:** 2022-03-04

**Authors:** Martin Anegg, Julia Prakofjewa, Raivo Kalle, Renata Sõukand

**Affiliations:** 1 Department of Environmental Sciences, Informatics and Statistics, Ca’ Foscari University of Venice, Mestre, Venezia VE, Via Torino, 155, 30170, Italy; 2 University of Gastronomic Sciences, Pollenzo CN, Piazza Vittorio Emanuele II, 9, 12042, Italy

**Keywords:** Ethnobotany, History, Folk Medicine, Aronson, Estonia, Livonia, Courland, Galicia

## Abstract

**Background:** Historical ethnobotanical data can provide valuable information about past human-nature relationships as well as serve as a basis for diachronic analysis. This data note aims to present a dataset which documented medicinal plant uses, mentioned in a selection of German-language sources from the 19 ^th^ century covering the historical regions of Estonia, Livonia, Courland, and Galicia.

**Methods:** Data was mainly obtained by systematic manual search in various relevant historical German-language works focused on the medicinal use of plants. Data about plant and non-plant constituents, their usage, the mode of administration, used plant parts, and their German and local names was extracted and collected into a database in the form of Use Reports.

## Plain language summary

This data note is based on a dataset ( Anegg *et al.,* 2021) which described medicinal plant uses in the 19th century in historical Estonia, Livonia, Courland and Galicia. The presented dataset is based on a digitized collection of German texts will be helpful to researchers who study the history of knowledge, science, and medicine.

## Introduction

Recent studies are underlining the diverse application possibilities of historical ethnobotanical research and the recent value of ethnobotanical data. The analysis of such data can contribute to understanding in which cultural fields plants are important and used, offer a rich basis of information for ethnobotanical and diachronic research, helping to understand better how societies and their folk culture develop and change over time, their dealing with natural resources, their interaction with and influence on ecosystems and the flora, as well as help in understanding modern medicinal practices better and contribute to the approval of new herbal medicines.

## Methods

The primary sources included in the presented database were identified through literature research focusing on local medicinal plant use in the historical regions of Estonia, Courland, Livonia, and Galicia. In addition, we included in the sample publications published between 1805 and 1905 solely in the German language as inclusion criteria ( Table 1). A range of relevant books and articles were used for extracting the historical indications of medicinal plant taxa which are treated as inclusion criteria (accessible botanical, historical, ethnographic literature describing the use of plants for medicinal purposes). Certain categories of sources are excluded from the dataset because of their non-circulating status (e.g., rare books). Main sources included the online libraries of the Online Catalogue ESTER ( Estonian Library Network Consortium), the Biodiversity Heritage Library ( Biodiversity Heritage Library), the Baltic Digital Library ( Bałtycka Biblioteka Cyfrowa) and google scholar, as well as citations and mentions of other relevant German-speaking authors (searching for documents which possess keywords: “Volksmedizin”, “Volksheilmittel”, “Heilpflanzen”, “Oekonomisch-technische Flora”). The limited geographical and temporal scale allows conducting a comprehensive comparative study to understand the biocultural diversity of medicinal ethnobotany of the region and to create a sound scientific base for future comparisons with current field-work results from the region.

**Table 1.** List of German language sources used in this study ( Anegg *et al.,* 2021).

Reference	Author	Title	Place of publication
Friebe, 1805	Friebe, Wilhelm Christian	Oekonomisch-technische Flora von Liefland, Ehstland und Kurland	Riga
Luce, 1829	Luce, Johann Wilhelm Ludwig von	Heilmittel der Ehsten auf der Insel Oesel	Pernau
Hoelzl, 1861	Hoelzl, Karl	Botanische Beiträge aus Galizien	Vienna
Wiedemann, 1876	Wiedemann, Ferdinand Johann	Aus dem inneren und äusseren Leben der Ehsten	Vienna
Aaronson, 1891	Aaronson, Dr. Emil	Über die Volksheilmittel der Letten	Mitau
Alksnis, 1894	Alksnis, Jakobs	Materialien zur lettischen Volksmedizin	Dorpat
Bermann & Ludwig, 1904	Bermann, P. und Ludwig, Mag. Pharm. F.	Pflanzen des Rigaschen Krautinarktes	Riga
Ludwig, 1905	Ludwig, Mag. Pharm. F.	Die Heilpflanzen des Rigaschen Krautmarktes	Riga

Due to the limited relevant written records in German language, every possible work was considered at first. For analysis purposes, we excluded primary sources that did not fulfil the following criteria: availability of local names and specific historical periods. In the next step, the selected public-domain books were carefully scanned and then was put into a Microsoft Excel 2013 spreadsheet. Thus, data on the local ecological knowledge and folk medicine were compiled from eight German historical ethnobotanical studies conducted in Estonia, Livonia, Courland, and Galicia, published between 1805 and 1905 ( Table 1).

Every independent use in the sources was considered as a use instance (UI) and was entered into a separate row in the spreadsheet. For each usage mentioned, the following information was elicited from the text, if present:

A. source
B. page number, where UI can be found
C. constituent type
D. constituent name stated in the original source
E. original German name of constituent, if provided
F. recent English interpretation
G. local name(s) of the constituent
H. preparation of constituent
I. plant part used (if applicable)
J. mode of administration
K. original usage of constituent
L. recent interpretation of medicinal usage
M. medicinal category (according to WHO, 2018)
N. recent interpretation of food usage
O. recent interpretation of other usage
P. additional comments

Besides recording all the medicinal usages of the different plants stated, information on other usages like food or veterinary medicinal uses were transcribed from the chosen texts and books as thoroughly as possible to allow further data mining and comparison possibilities in future studies. Moreover, non-plant constituents were transcribed for the same reason stated above.

In an additional step, important categories for analysis and future comparison with data from other investigations were unified according to the classifications used in other ethnobotanical and ethnomedicinal studies to facilitate comparisons with similar datasets.

These categories are

Q. plant name according to Plants of the World Online (POWO) ( POWO, 2021)
R. plant family current
S. medicinal use according to the International Classification of Primary Care (ICPC-2) ( WHO, 2012)
T. medicinal category short according to ICPC-2 ( WHO, 2012)
U. medicinal category abbreviation according to ICPC-2 ( WHO, 2012)

If an identification or accurate interpretation of a given constituent or any information of one of the categories stated above was not possible, the respective information was marked with a question mark in brackets ‘(?)’. Items with such a marking were excluded from the analysis.

The stated plant parts that were used were categorised as follows (with their respective abbreviation in square brackets): bark [BARK], exudates (including gums, resins, and saps) [EXUD], flowers (including inflorescences and parts thereof) [FLOW], fruits [FRUI], herbs (= aerial parts, including branches and shoots) [HERB], leaves [LEAV], seeds [SEED], subterranean parts (including bulbs, rhizomes, roots, and tubers) [SUBT] and wood [WOOD]. If the part used was not stated, then the part was classified as herbs. This categorisation follows the terminology used by the authors contributing to this study. Statements concerning “die Pflanze” (the plant) or “Grünzeug“ (greens) were also classified as herbs. Otherwise, the parts stated by the authors were the same in English terms, hence the categorisation. Furthermore, other studies, like Staub *et al.* (2016) and Spałek *et al.* (2019), also used this categorisation.

The mode of administration was recorded and divided into either internally (internal ingestion in any manner) or externally (for example, in the form of ointments or compresses) administered.

The recent interpretation of the ailments stated was done according to the International Statistical Classification of Diseases and Related Health Problems, Version 11 (ICD-11) of the World Health Organisation (WHO) ( WHO, 2018). This classification is divided into the following ailment categories:

1 - Certain infectious or parasitic diseases

2 - Neoplasms

3 - Diseases of the blood or blood-forming organs

4 - Diseases of the immune system

5 - Endocrine, nutritional or metabolic diseases

6 - Mental, behavioural or neurodevelopmental disorders

7 - Sleep-wake disorders

8 - Diseases of the nervous system

9 - Diseases of the visual system

10 - Diseases of the ear or mastoid process

11 - Diseases of the circulatory system

12 - Diseases of the respiratory system

13 - Diseases of the digestive system

14 - Diseases of the skin

15 - Diseases of the musculoskeletal system or connective tissue

16 - Diseases of the genitourinary system

17 - Conditions related to sexual health

18 - Pregnancy, childbirth or the puerperium

19 - Certain conditions originating in the perinatal period

20 - Developmental anomalies

21 - Symptoms, signs or clinical findings, not elsewhere classified

22 - Injury, poisoning or certain other consequences of external causes

X - Extension Codes (for example, for agents)

The unification of the recent interpretation of the ailments was carried out in accordance with the ICPC-2 of the World Organization of National Colleges, Academies and Academic Associations of General Practitioners/Family Physicians (WONCA) International Classification Committee ( WHO, 2012). This classification consists of the following categories (with the respective abbreviations used by the authors in the database and analysis in square brackets):

A - General and Unspecified diseases [Geun]

B - Blood, Blood Forming Organs and Immune Mechanism [Blim]

C - Culture Bound Syndrome (CultB)

D - Digestive [Dige]

F - Eye [Eye]

E - Ear [Ear]

K - Cardiovascular [Card]

L - Musculoskeletal [Musc]

N - Neurological [Neur]

P - Psychological [Psyc]

R - Respiratory [Resp]

S -Skin [Skin]

T - Endocrine/Metabolic and Nutritional [Endo]

W - Pregnancy, Childbearing, Family Planning [Pcfp]

X - Female Genital [Geni]

Y - Male Genital [Geni]

Z - Social Problems [Soci]

Those categories were segmented further into symptoms/complaints, infections, neoplasms, injuries, congenital anomalies, and other diagnoses. This classification was used for further analysis. This ICPC-2 categorisation was used for further analysis because it will facilitate easier comparison with other studies in the future. Furthermore, the ICPC-2 is less clinical than the ICD, making the classification of reported ailments and symptoms easier and more applicable to the ‘ethnomedical reality’ ( Staub *et al.,* 2015; Staub *et al.,* 2016). The categories ‘X - Female Genital’ and ‘Y - Male Genital’ were combined into one group ‘Genital’ [Geni] because almost no records concerning diseases of male genitalia were made by any of the contributing authors. The category of ‘culture bound syndrome’ was added to reflect the uses associated with local customs and beliefs not attributable to the specific disease categories.

Additional categories were added by the authors to cover non-medicinal usages. They are as follows:

a) “Accessories and Decoration” [ACDE] – including usages like wreaths, added to bouquets, etc.

b) “Body” [BODY] – including usages for body hygiene, restoring hair, baths generally, etc.

c) “Food” [FOOD] – including usages of plants as food or in food and beverages.

d) “Harmful” [HARM] – including reports of poisonous plants or usages to kill someone.

e) “Insecticides” [INSE] – including usages as an insecticide or to drive away insects.

f) “Other” [OTHE] – including all usages which do not fit into any of the other categories.

h) “Veterinary” [VETE] – including veterinary-medicinal usages concerning animals and pets.

i) “Cultural” [CULT] – including culture-bound usages of plants in a specific cultural setting.

To avoid misidentifications and misinterpretations of plants or historical technical medicinal terms, several sources were used for crosschecking past pathologies and plant names, including the Atlas of the Estonian Flora (2020), Beiche (1872), the GenWiki of the Verein für Computergenealogie e.V. (2020), GBIF.org (2021), Genaust (2013), Hiller & Melzig (2006), POWO (2021) and Tutin *et al.* (1993).

Disclaimer: the database is designed to give a general overview of the sources to the best knowledge of the authors. In case of any need for clarification, consult the original source.

## Data availability

### Underlying data

Zenodo. Local Ecological Knowledge and folk medicine in historical Esthonia, Livonia, Courland and Galicia, 1805–1905. https://doi.org/10.5281/zenodo.6106746. ( Anegg *et al.,* 2021).

This project contains the following underlying data:

- Anegg *et al.*_Database.xlsx. (An excel database was created by manually selecting relevant information and putting it into the database. Every independent use in the sources was accounted for as Detailed Use Reports (DUR), where the informant mentions specific medicinal use based on the category uses by the specific author of the plant part (p, e.g., fruits, leaves, aerial parts, flowers, etc. if provided), considering also the form in which the plant part is used (f, e.g., fresh, dried, frozen, refrigerated) and specific way of preparation. Every DUR was entered on a separate row in the excel spreadsheet).

Data are available under the terms of the Creative Commons Attribution 4.0 International license (CC-BY 4.0).
