# Supplementary material for: Local ecological knowledge and folk medicine in historical Estonia, Livonia, Courland and Galicia in Northeastern Europe, 1805-1905
Source: Open Res Eur. 2022 Sep 13;2:30. Originally published 2022 Mar 4. [Version 2] doi: 10.12688/openreseurope.14406.2 (PMC10445900; doi:10.12688/openreseurope.14406.2)
Supplement: Peer review history

# Peer review: Reviewer response for version 2

**DOI:** 10.21956/openreseurope.16292.r30083

**Published:** 2022-10-11

**Authors:** Irene Teixidor-Toneu

**Affiliations:** 1 Natural History Museum, University of Oslo, Oslo, Norway; 2 Centre d'Ecologie Fonctionnelle et Evolutive, CNRS, Paris, France

The map of the study area is a great addition. Please do indicate which area/colour corresponds to which named region either by adding the names in the map or explaining this in the figure legend.

 Something is still unclear to me. Are the four regions of study pre-selected (in which case, the question arises as to why this specific selection of regions and not other parts of Eastern Europe) or the four regions result from the search of data on herbal medicines in German in Eastern Europe (as in, there is only 19th century information in German in Eastern Europe for these four regions)?

 In the list of information present in the database, point B cites an acronym (UI) that is not explained.

Are sufficient details of methods and materials provided to allow replication by others?

Partly

Is the rationale for creating the dataset(s) clearly described?

Partly

Are the datasets clearly presented in a useable and accessible format?

Partly

Are the protocols appropriate and is the work technically sound?

Yes

Reviewer Expertise:

ethnopharmacology, ethnobotany, historical ethnobotany, biocultural approaches to conservation, evolutionary anthropology

I confirm that I have read this submission and believe that I have an appropriate level of expertise to confirm that it is of an acceptable scientific standard.

# Peer review: Reviewer response for version 1

**DOI:** 10.21956/openreseurope.15548.r29085

**Published:** 2022-05-09

**Authors:** Irene Teixidor-Toneu

**Affiliations:** 1 Natural History Museum, University of Oslo, Oslo, Norway; 2 Centre d'Ecologie Fonctionnelle et Evolutive, CNRS, Paris, France

This is a relevant and highly valid report on a dataset. I have made comments to improve the rationale for creating the database, as some gaps exist at present. I find the protocols appropriate and the work technically sound. Some details could be added to the methods and materials section and I have made suggestions to improve its usability and presentation.

 Abstract

 “historical regions of Estonia, Livonia, Courland, and Galicia” – it’s too vague. Galicia is also a region in Spain, but given that the documents are in German, I find it confusing. Add perhaps the state these historical regions are in brackets? Or the general region e.g., Eastern Europe? Baltics? In the introduction, a couple of sentences describing the overall or specific regions would be welcome.

 “Data was mainly obtained” – how were data not obtained by systematic manual search?

 “Various relevant historical German-language works” – how many? Relevant in what sense? Perhaps just delete the word “relevant”?

 Keywords

 I would suggest using words not present in the title to improve visibility of the paper in searches.

 Introduction

 “recent value of ethnobotanical data” – I disagree that the value is “recent”, perhaps re-valorised?

 Methods

 It is not clear why these regions were chosen (a map would be most welcome), why the specified time period, or why focus on German language sources. These questions could perhaps be addressed in the introduction. Are sources in other languages about these regions already systematised? Or are there plans to do so?

 Does a description of each source accompany the database? Interpretation of medicinal plant use documented in historical sources heavily relies on understanding the author’s motivations and context of production of the source. Such information would be necessary for a broader use of the dataset.

 In the methods “Use Instance” is used instead of “Use Report”, which is noted in the abstract. It would be good to be consistent with one or other phrasing.

 What is the difference between “constituent name stated in original” and “local names”?

 The inclusion of food and veterinary uses is highly welcome, for the purposes stated by the authors.

 “Items with such a marking were excluded from the analysis” – I wonder if this sentence belongs to this text. As far as I understand, no analysis is expected in an Open Research Europe Data Note.

 For the database to be fully expandable, I would suggest not to combine categories, even if some are not found in the current dataset (e.g., X – Female Genital, Y- Male Genital).

 For the dataset to be easily usable, it should also include a tab with the codes used. I am aware that these can be found in associated publications, but including them in the dataset itself will facilitate further use.

Are sufficient details of methods and materials provided to allow replication by others?

Partly

Is the rationale for creating the dataset(s) clearly described?

Partly

Are the datasets clearly presented in a useable and accessible format?

Partly

Are the protocols appropriate and is the work technically sound?

Yes

Reviewer Expertise:

ethnopharmacology, ethnobotany, historical ethnobotany, biocultural approaches to conservation, evolutionary anthropology

I confirm that I have read this submission and believe that I have an appropriate level of expertise to confirm that it is of an acceptable scientific standard, however I have significant reservations, as outlined above.

# Response

**Published:** 2022-08-25

**Authors:** Prakofjewa Julia

**Affiliations:** Ca' Foscari University of Venice, Italy

*This is a relevant and highly valid report on a dataset. I have made comments to improve the rationale for creating the database, as some gaps exist at present. I find the protocols appropriate and the work technically sound. Some details could be added to the methods and materials section, and I have made suggestions to improve its usability and presentation. Response: Thank you very much for your careful reading and helpful comments and suggestions.  Abstract “historical regions of Estonia, Livonia, Courland, and Galicia” – it’s too vague. Galicia is also a region in Spain, but given that the documents are in German, I find it confusing. Add perhaps the state these historical regions are in brackets? Or the general region e.g., Eastern Europe? Baltics? In the introduction, a couple of sentences describing the overall or specific regions would be welcome.*

**Response:** We are very grateful to the reviewer for this comment. We have been more specific in the title, modifying it as follows.  “Local ecological knowledge and folk medicine in historical Estonia, Livonia, Courland and Galicia in Northeastern Europe, 1805-1905”.  We have clarified it in the introduction as follows: “This data note is based on a dataset (Anegg *et al*., 2021) which described medicinal plant uses in the 19th century in historical Estonia, Livonia, Courland and Galicia, which were located in Northeast Europe. The studied region corresponds roughly to present-day Latvia (Livonia and Courland), Estonia and Ukraine (Galicia).”

*“Data was mainly obtained” – how were data not obtained by systematic manual search? “Various relevant historical German-language works” – how many? Relevant in what sense? Perhaps just delete the word “relevant”?*

**Response:** Thank you to the reviewer for the careful reading. We have corrected it as follows.

 “Methods: Data was mainly entered by systematic manual search in various ethnobotanical historical German-language works focused on the medicinal use of plants.”

*Keywords I would suggest using words not present in the title to improve visibility of the paper in searches*.

**Response:** Thank you very much for your comment; we further specified the keywords as follows:  “Keywords: ethnomedicine, historical ethnobotany, Baltics, environmental history, herbals”

*Introduction “recent value of ethnobotanical data” – I disagree that the value is “recent”, perhaps re-valorised?*

**Response:** Thank you very much for bringing this to our attention. We have further corrected as follows. “Recent studies are underlining the diverse application possibilities of historical ethnobotanical research and the re-valorised value of ethnobotanical data.”

*Methods It is not clear why these regions were chosen (a map would be most welcome), why the specified time period, or why focus on German language sources. These questions could perhaps be addressed in the introduction. Are sources in other languages about these regions already systematised? Or are there plans to do so?*

**Response:** We are grateful for these comments. We have added the map of the studied region in the Methods.  We have further expanded, referring to previous articles published by members of our research group as follows.  “The research was part of a wider study, namely the ERC-funded DiGe project, aiming to understand the patterns of change in ethnobotanical knowledge systems in Eastern European countries. Specifically, we have selected the period and space to fill the gap in historical ethnobotanical studies. German language was the dominant language of scientific communication in the studied period. So German language herbals devoted to Baltics were not studied from ethnobotanical point of view yet. The growing interest in the historical ethnobotany of Eastern Europe may be observed - it was already have done a dataset of medicinal plants, based on written sources in Estonian (Soukand *et al* 2008). From historical point of view the ethnobotanical data affecting studied region was already systematised and analyzed by Estonian scholars (Kalle *et al* 2022, Kalle *et al* 2021)”.  

 We have added the references as follows: Kalle R, Sõukand R. The name to remember: Flexibility and contextuality of preliterate folk plant categorization from the 1830s, in Pernau, Livonia, historical region on the eastern coast of the Baltic Sea. J Ethnopharmacol. 2021 Jan 10;264:113254. doi: 10.1016/j.jep.2020.113254. Kalle R, Pieroni A, Svanberg I, Sõukand R. Early Citizen Science Action in Ethnobotany: The Case of the Folk Medicine Collection of Dr. Mihkel Ostrov in the Territory of Present-Day Estonia, 1891–1893. Plants. 2022; 11(3):274. https://doi.org/10.3390/plants11030274 Sõukand R, Kalle R. Historistlik eesti rahvameditsiini botaaniline andmebaas (HERBA). [HERBA, the Estonian folk medicine database of plant use. 2008]. We have corrected it as follows: 1. Aaronson E: Ueber die Volksheilmittel der Letten. Magazin der Lettischliterärischen Gesellschaft. ed. A. Bielenstein, Mitau, 1891; 19(1): 185–203

*Does a description of each source accompany the database? Interpretation of medicinal plant use documented in historical sources heavily relies on understanding the author’s motivations and context of production of the source. Such information would be necessary for a broader use of the dataset.*

**Response:** Thank you, we have specified it referring to previous articles published by our group as follows. “A more detailed analysis of medicinal plant records documented in the dataset was presented in the paper (Prakofjewa et al 2022).” We have added the reference as follows: Prakofjewa J, Anegg M, Kalle R, Simanova A, Prūse B, Pieroni A, Sõukand R. Diverse in Local, Overlapping in Official Medical Botany: Critical Analysis of Medicinal Plant Records from the Historic Regions of Livonia and Courland in Northeast Europe, 1829–1895. Plants. 2022; 11(8):1065. https://doi.org/10.3390/plants11081065

*In the methods “Use Instance” is used instead of “Use Report”, which is noted in the abstract. It would be good to be consistent with one or other phrasing.*

**Response:** Thank you for your note. We have corrected it as follows. “Every independent use in the sources was considered as a Use Report (UR) and was entered into a separate row in the spreadsheet”

*What is the difference between “constituent name stated in original” and “local names”?*

**Response:** Thank you for bringing this to our attention. We have further expanded as follows. “It should be noted that the “constituent name stated in original” reported plant names which are stated in German and Latin languages, but “local name” is always stated as indigenous name (if recorded)”. 

*The inclusion of food and veterinary uses is highly welcome, for the purposes stated by the authors.*

**Response: **Thank you for noticing it. It contains all the uses mentioned in the sources. 

*“Items with such a marking were excluded from the analysis” – I wonder if this sentence belongs to this text. As far as I understand, no analysis is expected in an Open Research Europe Data Note.*

**Response:** Thank you for noticing. We have deleted it.

*For the database to be fully expandable, I would suggest not to combine categories, even if some are not found in the current dataset (e.g., X – Female Genital, Y- Male Genital).*

**Response:** Thank you, we have further expanded as follows. “Despite the fact that in the documented historical sources it was not found special categories (e.g., Y- Male Genital), we kept the ICPC-2 categorisation for future perspective of the development of the database and adding new German-speaking authors.”

*For the dataset to be easily usable, it should also include a tab with the codes used. I am aware that these can be found in associated publications, but including them in the dataset itself will facilitate further use.*

**Response:** Thank you for this idea which contributes to effectively deliver the information of this Data Note. We have developed the following dataset (Anegg et al 2021).

# Peer review: Reviewer response for version 1

**DOI:** 10.21956/openreseurope.15548.r28720

**Published:** 2022-03-21

**Authors:** Inga Sile

**Affiliations:** 1 Latvian Institute of Organic Synthesis, Riga, Latvia

This data note describes medicinal plant uses (also usages in food or veterinary) in the 19th century in historical Estonia, Livonia, Courland and Galicia. A systematic search has been conducted to collect relevant historical German-language works focused on the medicinal use of plants. Collected data are well structured in the database which can be useful to other researchers, especially those studying ethnobotany and ethnomedicine.

 The rationale for the creation of the dataset is clearly described. The work is well organized and technically sound. This data note provides easy-to-understand and sufficient information about the methods and materials so that others can successfully replicate them. An excel database is clearly understandable and easy to use for searching or selecting specific information.

 The work ‘Local ecological knowledge and folk medicine in historical Estonia, Livonia, Courland, and Galicia, 1805-1905’ is well performed and can be useful to analyze the development of folk culture, the traditions of folk medicine and changes over time. I have only one remark: the inclusion of such historical regions as Estonia, Livonia, and Courland sounds justified, but I am not sure if Galicia falls well within the mentioned regions. Perhaps you should add a sentence explaining why these regions have been chosen.

Are sufficient details of methods and materials provided to allow replication by others?

Yes

Is the rationale for creating the dataset(s) clearly described?

Yes

Are the datasets clearly presented in a useable and accessible format?

Yes

Are the protocols appropriate and is the work technically sound?

Yes

Reviewer Expertise:

My areas of research are medicinal plant uses, pharmacognosy, ethnobotany, ethnomedicine, analysis of chemical composition of plant material and biological activity analysis in vitro and ex vivo.

I confirm that I have read this submission and believe that I have an appropriate level of expertise to confirm that it is of an acceptable scientific standard.
